# The Effects of *AtNCED3* on the Cuticle of Rice Leaves During the Nutritional Growth Period

**DOI:** 10.3390/ijms26146690

**Published:** 2025-07-12

**Authors:** Yang Zhang, Yuwei Jia, Hui Chen, Min Wang, Xiaoli Li, Lanfang Jiang, Jianyu Hao, Xiaofei Ma, Hutai Ji

**Affiliations:** 1 Wheat Research Institute, Shanxi Agricultural University, Linfen 041000, China; yangzhang610@163.com (Y.Z.); wangmin5322@163.com (M.W.); lixiaoli617@163.com (X.L.); jianglf1013@163.com (L.J.); 18735429168@163.com (J.H.); 2 College of Life Sciences, Shanxi Normal University, Taiyuan 030000, China; jiayuwei610@163.com; 3 College of Agriculture, Northwest A&F University, Yangling 712100, China; hc91830709@163.com

**Keywords:** *AtNCED3*, ABA, vegetative growth period, rice, cuticle

## Abstract

The plant cuticle, a protective barrier against external stresses, and abscisic acid (ABA), a key phytohormone, are crucial for plant growth and stress responses. Heterologous expression of *AtNCED3* in plants has been widely studied. In this research, by comparing the japonica rice cultivar Zhonghua 10 and its *AtNCED3* over-expressing lines during the vegetative growth stage through multiple methods, we found that *AtNCED3* over-expression increased leaf ABA content, enhanced epidermal wax and cutin accumulation, modified wax crystal density, and thickened the cuticle. These changes reduced leaf epidermal permeability and the transpiration rate, thus enhancing drought tolerance. This study helps understand the role of endogenous ABA in rice cuticle synthesis and its mechanism in plant drought tolerance, offering potential for genetic improvement of drought resistance in crops.

## 1. Introduction

Plants face diverse stresses from natural environments, including drought, low temperatures, UV radiation, and pests [1]. The plant cuticle, a multifunctional protective layer on aerial plant parts, acts as the first defense against these stresses [2]. Comprising an inner cutin polyester matrix and outer waxy substances, the cuticle’s hydrophobic nature helps plants combat abiotic and biotic challenges [3,4,5,6,7]. Cutin, its structural backbone, consists of fatty acids and derivatives, while waxy substances, mainly very long-chain fatty acids (VLCFAs) and related compounds, are divided into intracuticular and epicuticular wax, with the latter forming surface crystals [3,8,9]. The epidermal cuticle’s importance in plant functionality has drawn growing research interest.

Abscisic acid (ABA), a major plant hormone, regulates growth and stress responses [10]. 9-cis-epoxycarotenoid dioxygenase (NCED) is pivotal in ABA biosynthesis [11]. ABA inhibits seed germination, and its levels drop during this process [12,13]. Previous studies showed that *AtNCED3* over-expression (OE) increases ABA content, enhancing drought resistance in soybeans, Arabidopsis, and rice, and altering the cuticle, suggesting that ABA impacts cuticle biosynthesis [14,15].

Despite numerous reports on exogenous ABA’s effects on the plant cuticle, the role of endogenous ABA in rice cuticle remains unexplored. This study used japonica rice Zhonghua 10 (CK) and *Ubi1::AtNCED3* L4 transgenic lines to analyze leaf physiology, microstructures, cuticle wax, and related gene expression. Our goal was to clarify how endogenous ABA affects rice cuticle synthesis, laying the groundwork for understanding ABA’s role in plant drought resistance.

## 2. Results

### 2.1. Identification of Over-Expressing Plants

**Figure 1:**
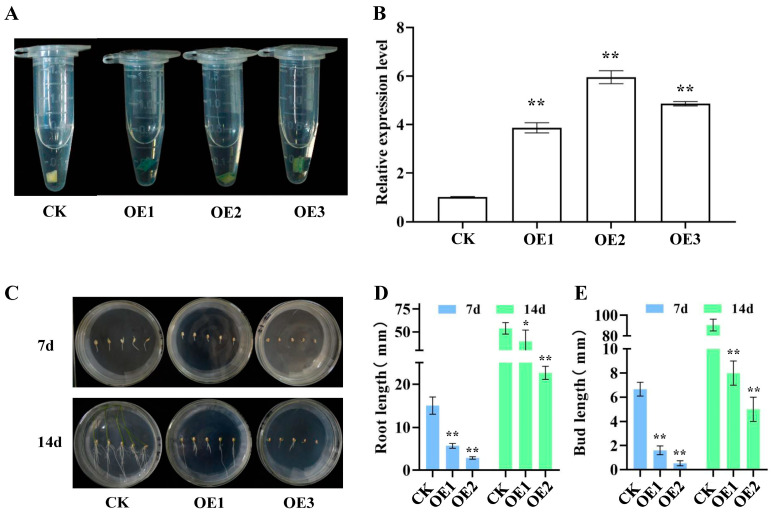
Screening of over-expressing plants. (**A**). GUS histochemical staining. (**B**). Relative expression analysis of *Ubi1::AtNCED3*. (**C**). Germination of seeds. (**D**,**E**). Measurement of root length and sprout length. Significant differences among different comparisons were determined with Duncan’s multiple range test, and significant and highly significant differences are indicated by * (*p* < 0.05) and ** (*p* < 0.01), respectively.

The β-glucuronidase (GUS) staining indicated that the *Ubi1::AtNCED3* construct was successfully integrated into OE1, OE2, and OE3 plants, resulting in over-expressed leaves turning blue, whereas CK leaves remained colorless (Figure 1A). Real-time quantitative PCR (qPCR) analysis confirmed that *AtNCED3* expression was significantly higher in the OE lines compared to the CK (Figure 1B), leading to the subsequent selection of OE1 and OE3 for further investigation. Measurements of tissue-cultured seedlings demonstrated that at 7 and 14 days, OE1 and OE3 exhibited shoots that were 81.43% to 91.43% shorter and roots that were 64.71% to 82.35% shorter than those of the CK (Figure 1C–E), suggesting that *AtNCED3* may inhibit seed germination.

### 2.2. Altered Phenotypes in AtNCED3 Over-Expressing Rice Plants

**Figure 2:**
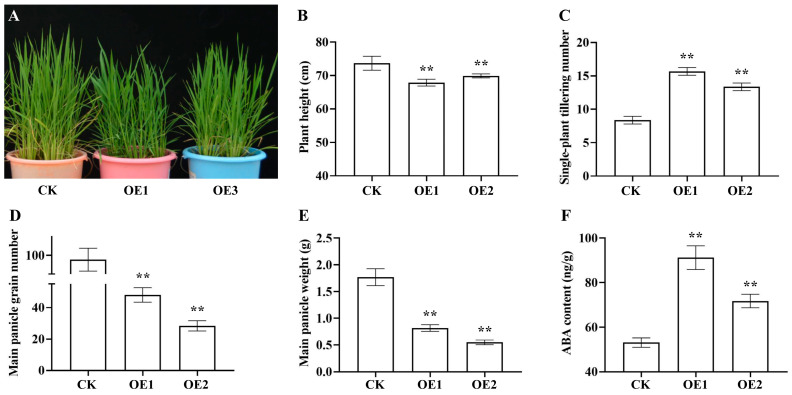
The phenotypic observation of rice. (**A**). Phenotypic observation. (**B**). Plant height. (**C**). Single plant tillering number. (**D**). Main panicle grain number. (**E**). Main panicle weight. (**F**). ABA content. Significant differences among different comparisons were determined with Duncan’s multiple range test, and significant and highly significant differences are indicated by ** (*p* < 0.01).

During the vegetative growth stage, lines over-expressing *AtNCED3* exhibited a dwarf phenotype characterized by dark green, broad, and thick leaves (Figure 2A). Plant height was reduced by 7.92% and 5.16% relative to the CK (Figure 2B and Table S1), whereas the number of tillers increased by 88.00% and 60.00% (Figure 2C and Table S1).

Upon reaching maturity, the CK had 97 grains per main panicle and a panicle weight of 1.77 g, compared to 48/28 grains and 0.82/0.55 g for OE1/OE3 (Figure 2D,E and Table S1). The ABA content in the over-expression lines was 71.90% and 35.24% higher than in the CK (Figure 2F and Table S1). These findings suggest that the over-expression of *AtNCED3* increases ABA content, thereby altering the growth and development of rice.

### 2.3. Water-Holding Capacity of AtNCED3 Over-Expressing Rice Leaves

**Figure 3:**
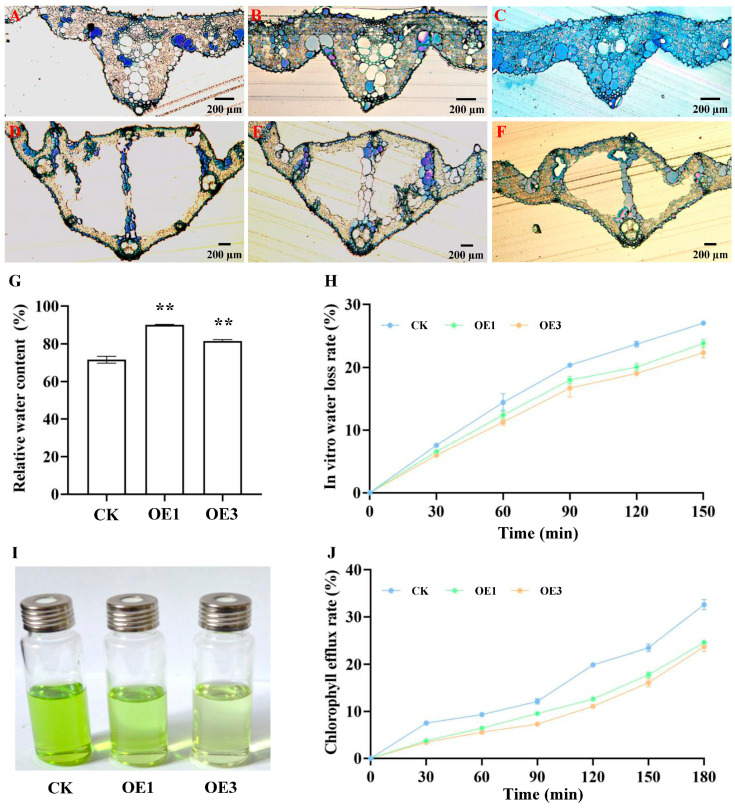
Leaf water retention. (**A**). The leaf of CK. (**B**). The leaf of OE1. (**C**). The leaf of OE3. (**D**). The leaf Veins of CK; (**E**). The leaf veins of OE1; (**F**). The leaf veins of OE3; (**G**). Relative water content of leaves; (**H**). In vitro water loss rate; (**I**,**J**). The chlorophyll efflux rate. Significant differences among different comparisons were determined with Duncan’s multiple range test, and significant and highly significant differences are indicated by ** (*p* < 0.01).

Cross-sections of rice leaves and veins revealed that lines over-expressing *AtNCED3* had larger and more numerous bubble cells and smaller intercellular spaces (Figure 3A–F), which resulted in a reduced stomatal size compared to the CK. Relative water content (RWC) measurements (Figure 3G) indicated that the over-expression lines had 25.72% and 13.93% higher water content. In vitro water loss rate assays (Figure 3H) showed lower water loss rates over 150 min, and chlorophyll efflux experiments (Figure 3I,J) demonstrated decreased rates within 180 min, with OE3 performing the best. These findings suggest that the over-expression of *AtNCED3* enhances leaf water-holding capacity, likely through ABA-induced cuticle thickening, reduced cell permeability, and decreased non-stomatal transpiration.

### 2.4. SEM Analysis of Epicuticular Wax Crystals on Rice Leaf Epidermis

**Figure 4:**
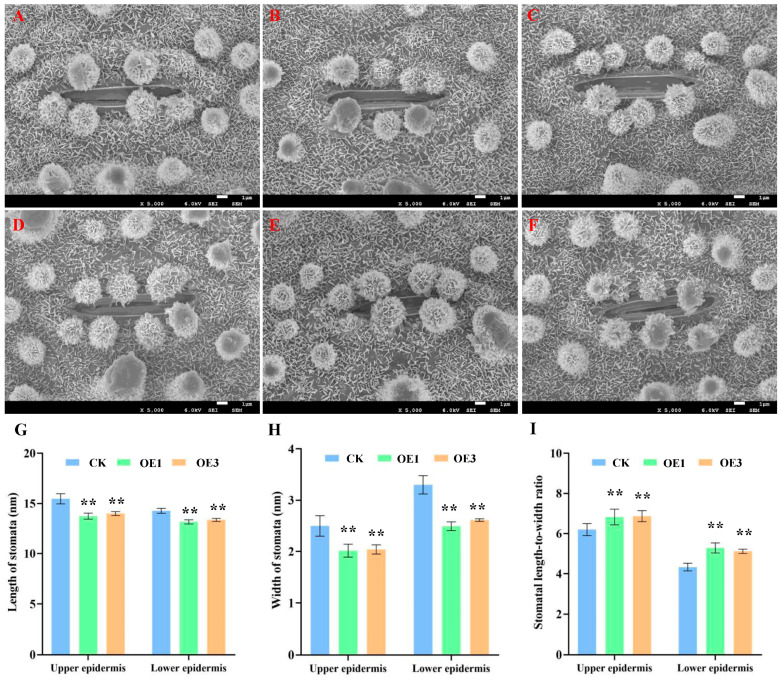
Electron micrograph of wax crystals in rice leaves. (**A**–**C**). Upper epidermis. (**D**–**F**). Lower epidermis (bar: 1 μm, ×5.0 K). (**G**). Length of stomata. (**H**). Width of stomata. (**I**). Stomata length-width ratio. Significant differences among different comparisons were determined with Duncan’s multiple range test, and significant and highly significant differences are indicated by ** (*p* < 0.01).

Scanning electron microscopy (SEM) observations (Figure 4A–F) revealed that over-expression lines exhibited a significantly higher density of epicuticular wax crystals with a more dendritic distribution, increased papillae around stomata, and reduced stomatal aperture and diameter on both leaf surfaces (Figure 4G–I and Table S2). These findings suggest that *AtNCED3* positively regulates the accumulation of epicuticular wax crystals.

In summary, the over-expression of *AtNCED3* enhances rice leaf water-holding capacity, likely through ABA-induced cuticle thickening, reduced cell permeability, and lower non-stomatal transpiration.

### 2.5. Changes in Composition and Content of Rice Leaf Epicuticular Wax

**Figure 5:**
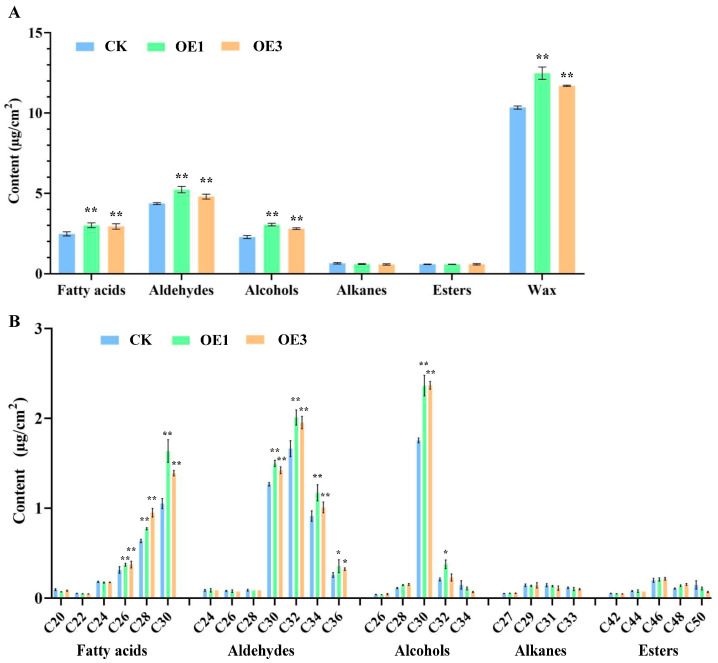
Analyses of wax contents and constituents in rice leaves. (**A**) Comparison of total contents of various wax components in rice leaves. (**B**) Comparison of contents of various compounds in epidermal wax of rice leaves. Significant differences among different comparisons were determined with Duncan’s multiple range test, and significant and highly significant differences are indicated by * (*p* < 0.05) and ** (*p* < 0.01), respectively.

Gas Chromatography–Mass Spectrometry (GC-MS) analysis of leaf epidermal wax from CK and over-expression lines showed that all contained substantial amounts of fatty acids, aldehydes, and alcohols (Figure 5A and Table S3). The total wax content in over-expression leaves was 21.95% and 13.87% higher than in CK. Further analysis revealed significant increases in C26, C28, and C30 fatty acids; C30, C32, and C34 aldehydes; and C30 alcohol in over-expression lines. However, alkane and ester levels showed no significant differences (Figure 5B and Table S3). Thus, *AtNCED3* expression primarily enhances the synthesis of fatty acids, aldehydes, and alcohols in wax components, with minimal impact on alkanes and esters.

### 2.6. Alterations in the Composition and Content of Rice Leaf Cutin Monomers

**Figure 6:**
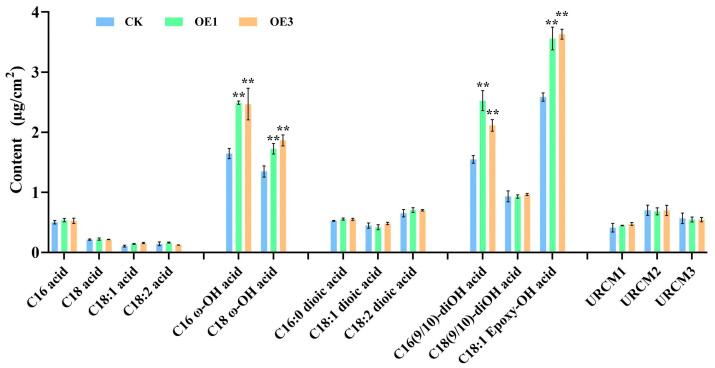
Analyses of cutin monomer composition and contents in rice leaves. Significant differences among different comparisons were determined with Duncan’s multiple range test, and significant and highly significant differences are indicated by ** (*p* < 0.01).

GC-MS analysis revealed that the total cutin monomer content in OE1 and OE3 was 27.0% and 25.8% higher than in CK, respectively (15.67, 15.52 vs. 12.34 μg/cm^2^; Figure 6 and Table S4). Key components, such as C16:0 ω-OH and C18 Epoxy-OH fatty acids, increased significantly, indicating *AtNCED3*’s role in cutin monomer hydroxylation. Overall, the over-expression of *AtNCED3* enhances leaf water-holding capacity through ABA-induced cuticle modification and reduced transpiration.

### 2.7. Transcription—Level Control of Wax and Cutin Biosynthesis—Related Genes in Rice Leaves

**Figure 7:**
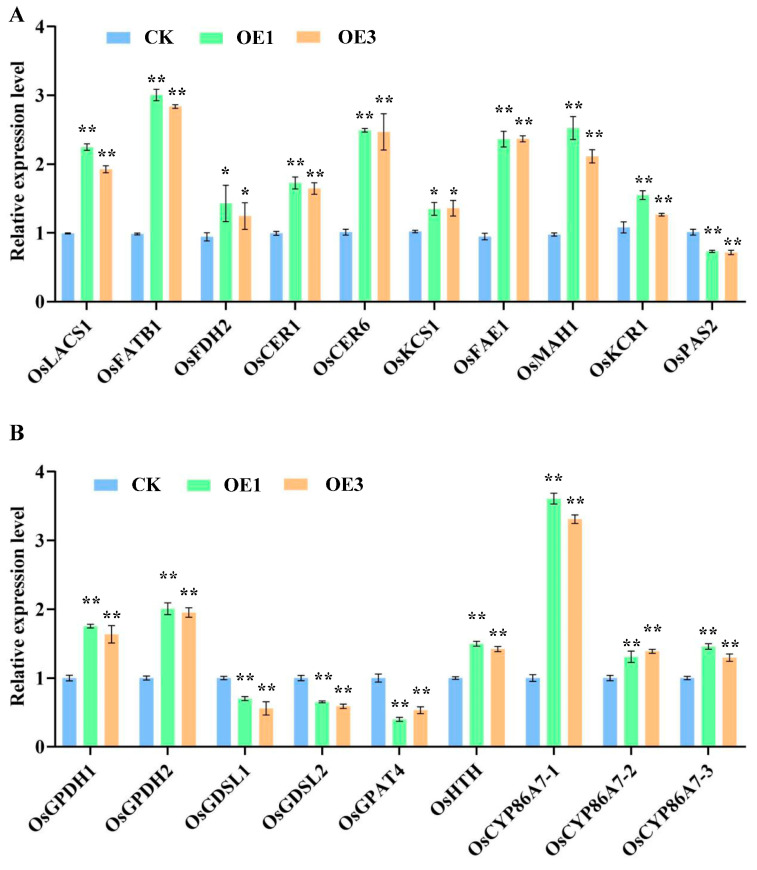
Expression profiles of wax and cutin synthesis-related genes in rice leaves. (**A**). Expression profiles of wax synthesis-related genes. (**B**). Expression profiles of cutin synthesis-related genes. Significant differences among different comparisons were determined with Duncan’s multiple range test, and significant and highly significant differences are indicated by * (*p* < 0.05) and ** (*p* < 0.01), respectively.

Real-time quantitative PCR analysis revealed that nine wax-associated genes (*OsLACS1*, *OsFATB1*, etc.) were upregulated in over-expression lines, with *OsCER6*, *OsFAE1*, *OsMAH1*, *OsLACS1*, and *OsFATB1* exhibiting significant increases. Specifically, *OsFATB1* expression increased by 3.05-fold in OE1 and 2.88-fold in OE3 (Figure 7A).

Regarding cutin-related genes, six genes (*OsGPDH1*, *OsCYP86A7-1*, etc.) were upregulated. Among these, *OsCYP86A7-1* showed the most substantial induction, with increases of 3.61-fold and 3.31-fold in OE1 and OE3, respectively (Figure 7B).

These results suggest that over-expression lines maybe enhance epidermal lipid biosynthesis by coordinately upregulating *OsFATB1* (wax pathway) and *OsCYP86A7-1* (cutin pathway), offering genetic targets for understanding plant cuticular barrier formation.

## 3. Discussion

### 3.1. The Impact of AtNCED3 on Rice Plant Architecture

ABA, a pivotal stress-responsive phytohormone, orchestrates plant adaptation to drought and other abiotic stresses by modulating multiple physiological pathways, including the metabolism of storage proteins and lipids in seeds [16,17]. 9-cis-Epoxycarotenoid dioxygenase, the rate-limiting enzyme in ABA biosynthesis, plays a central role in regulating ABA levels, with *AtNCED3* being a major determinant of ABA synthesis in Arabidopsis thaliana.

In the present study, ectopic expression of *Ubi1::AtNCED3* in rice significantly impacted plant growth and development. During germination, over-expression lines exhibited delayed growth, likely due to the inhibitory effect of elevated ABA levels on seed germination. This finding aligns with previous reports demonstrating that *AtNCED3* is critical for ABA production during early seed development; its over-expression enhances seed ABA accumulation and extends dormancy [18]. During the vegetative growth stage, *AtNCED3* over-expressing plants displayed a distinct phenotype characterized by a 6.53% reduction in plant height, a 74% increase in tiller number, and a 53.57% elevation in endogenous ABA content compared to CK. The observed decrease in plant height may result from *AtNCED3*-mediated growth suppression, which concurrently activates downstream signaling pathways. This activation promotes stomatal closure and stimulates the synthesis of osmoprotectants, leading to increased relative water content, reduced chlorophyll leakage, decreased transpiration rates, and enhanced water-holding capacity [19].

Notably, in the reproductive phase, *AtNCED3* over-expressing lines showed decreased seed-setting rates (60.92% of CK) and panicle weights (61.36% of CK). However, the higher tiller numbers in transgenic plants compensated for these reductions, resulting in a significantly increased thousand-grain weight relative to CK. These results are consistent with previous studies in soybean, where *AtNCED3* over-expression improved water-use efficiency and enhanced drought tolerance [15]. These results show that *AtNCED3* over-expression may enhance drought tolerance by significantly activating the ABA synthesis pathway and downstream drought-responsive genes. However, it is important to note that these conclusions regarding drought tolerance are based on indirect inferences from the observed physiological changes under normal growth conditions, as drought stress experiments were not conducted in the present study.

Collectively, our findings demonstrate that *AtNCED3* over-expression activates the ABA biosynthesis pathway and downstream drought-responsive genes in rice. These results provide a theoretical foundation for developing genetic strategies to mitigate yield losses under drought conditions, highlighting *AtNCED3* as a promising target for molecular breeding in rice. Future studies should include experiments under drought stress conditions to directly validate the effects of *AtNCED3* over-expression on drought tolerance in rice.

### 3.2. The Impact of AtNCED3 on the Rice Leaf Cuticle

In the cuticle biosynthesis pathway, C16 and C18 fatty acids are initially synthesized within plastids and subsequently transported to the cytoplasm, where they diverge into two distinct metabolic routes: wax and cutin biosynthesis [20]. During wax synthesis, these long-chain fatty acids serve as substrates for the multi-enzyme fatty acid elongase complex, which catalyzes their elongation into VLCFAs precursors, predominantly generating saturated VLCFAs with C24 to C36 [21]. These VLCFAs are then metabolized via two major pathways: the acyl reduction pathway, which produces primary alcohols and wax esters, and the decarbonylation pathway, responsible for the formation of aldehydes, alkanes, secondary alcohols, and ketones [9].

GC-MS analysis of rice leaf wax components revealed a profile consistent with previous findings by Zhou [22], primarily consisting of fatty acids, aldehydes, and alcohols. Notably, *AtNCED3* over-expression lines exhibited significantly elevated levels of these wax components compared to the CK. Transcriptional analysis further demonstrated upregulation of genes involved in VLCFA synthesis in leaves, including *OsLACS1*, *OsFATB1*, *OsFDH2*, *OsKCR1*, *OsFAE1*, and *OsCER6*. This suggests that *AtNCED3* over-expression activates early stages of wax metabolism, supplying substrates, especially for the decarbonylation pathway. Moreover, the expression of *OsCER1* and *OsMAH1*, key enzymes in the decarbonylation pathway, was also significantly enhanced, correlating with the increased C32 aldehyde content observed in transgenic lines. Specifically, *FATB1* (acyl-ACP thioesterase) [23] and *LACS1* (acyl-CoA synthetase) [24] initiate C16–C18 fatty acid activation for VLCFA synthesis, while *OsCER1* [25] and *OsMAH1* [26] mediate subsequent decarbonylation reactions. Four other genes encode essential subunits of the fatty acid elongase complex.

Cutin, a major cuticle component, comprises polyesters of C16 and C18 oxidized fatty acids and glycerol [27]. The biosynthesis of C16 cutin monomers begins with ω-hydroxylation of palmitic acid, forming 16-hydroxy palmitic acid (C16 ω-OH acid), followed by mid-chain hydroxylation to produce 9 (10)-16-di-hydroxy palmitic acid. For C18 cutin monomers, oleic acid undergoes sequential ω and mid-chain hydroxylation, followed by epoxidation to yield tri-hydroxy C18 monomers 4]. GC-MS analysis showed a substantial increase in cutin monomer content, particularly C16 and C18 ω-hydroxylated fatty acids, in the leaves of *AtNCED3* over-expression lines. These findings suggest that *AtNCED3* over-expression predominantly impacts the hydroxylation steps in cutin biosynthesis, specifically those involved in the formation of C16 and C18 ω-OH and C16 (9/10)-diOH fatty acids. Transcriptional data further support this hypothesis, as genes associated with hydroxylation, including *OsCYP86A7-1/2/3* and *OsGPDH1/2*, were significantly upregulated in transgenic plants. Collectively, these results indicate that *AtNCED3* plays a pivotal role in cuticle synthesis by regulating key hydroxylation reactions in cutin monomer formation.

While these results offer valuable insights into the role of *AtNCED3* in cuticle synthesis, it is important to note that the current study did not include experiments conducted under drought stress. Therefore, conclusions regarding the potential drought tolerance conferred by *AtNCED3* over-expression are based on indirect inferences drawn from changes in cuticle composition and physiological traits under normal growth conditions. Future studies should incorporate drought stress experiments to directly assess the effects of *AtNCED3* over-expression on drought tolerance. This will help to confirm whether the observed changes in cuticle and physiological traits indeed lead to enhanced drought resistance in field conditions.

### 3.3. Limitations and Future Prospects of This Study

Despite achieving certain results in exploring the effects of heterologous expression of *AtNCED3* on the leaf cuticle of rice during the vegetative growth stage, this study still has limitations. On the one hand, experiments under drought stress conditions were not conducted, which may fail to more comprehensively reveal the drought-resistant molecular mechanisms of *AtNCED3* in rice (Zhonghua 10). On the other hand, the phenotypes and mechanisms of over-expressing *AtNCED3* have not been verified in other rice genotypes. Subsequent studies can validate the findings through drought-related experiments to more comprehensively support the research results. The current results still provide valuable insights into the role of endogenous ABA in rice cuticle synthesis and its contribution to plant drought resistance. They also pave new ways for the genetic improvement of crop stress resistance. Utilizing this gene can lead to the development of new rice varieties with drought tolerance during the vegetative growth stage. Such sustainable varieties can reduce the consumption of agricultural resources, lower production costs, and alleviate environmental pressures, in line with the requirements of sustainable agricultural development.

## 4. Materials and Methods

### 4.1. Plant Materials

The seeds of the japonica rice cultivar Zhonghua 10 and the T_2_ generation of the transgenic *Ubi1::AtNCED3* line were kindly provided by Professor Chen Hui’s laboratory at Shanxi Normal University.

### 4.2. Methods

#### 4.2.1. Over-Expression Plant Identification

The T_2_ transgenic line seeds were surface-sterilized and germinated on MS medium for a duration of one month. Following this, leaf segments measuring 2–3 cm were excised, finely incised, and incubated in GUS staining solution at 37 °C for a period of 24 h. The leaf tissues were then transferred to 70% ethanol to remove chlorophyll, and the blue coloration indicating GUS expression was examined.

For genetic confirmation, leaf segments of 2–3 cm were used to extract genomic DNA using the Plant Genomic DNA Extraction Kit (TIANGEN, Beijing, China, DP305-03). PCR amplification was conducted using the following primers: Forward (5′-CTACCCGGATGGCTTCTTTACGGCAAAG-3′) and Reverse (5′-GTAGAGCTCTCACACGACCTGATTCGCCAAATC-3′), under these conditions: pre-denaturation at 94 °C for 2 min, 35 cycles of 98 °C for 10 s, 58 °C for 30 s, 68 °C for 1 min, a final extension at 68 °C for 7 min, and a hold at 16 °C. The 1800 bp PCR products were analyzed by 1% agarose gel electrophoresis to verify the integration of the *Ubi1::AtNCED3* transgene.

#### 4.2.2. Real-Time Quantitative PCR Analysis of Over-Expressing Plants

For gene expression analysis, 0.1 g of fresh leaves from PCR-positive plants was ground in liquid nitrogen, and total RNA was extracted using the Plant Total RNA Extraction Kit (TSINGKE TSP0201). After measuring the RNA concentration, quantitative PCR primers were designed with Primer 5 software. cDNA was synthesized from the RNA using the TaKaRa reverse transcription kit. With 18S rRNA as the endogenous reference and SYBR Green I as the dye, the expression levels of 25 wax- and cutin-related genes were quantified on an Applied Biosystems 7500 Real-Time PCR System (Thermo, Waltham, MA, USA). All reactions were performed in triplicate, and the primer sequences are listed in Table S5 [22]. Relative gene expression was calculated using the 2^−∆∆CT^ method [28].

#### 4.2.3. Phenotypic Observation During Germination

Seeds from the CK and the T_3_ over-expressing lines (OE1 and OE3) were surface-sterilized as follows: 3 min in 70% ethanol, followed by 2–3 rinses with sterile water, 13 min in 0.1% mercuric chloride, and finally, 4–5 rinses with sterile water. CK seeds were cultured in MS0 medium, whereas OE1 and OE3 seeds were placed in MS0 medium supplemented with 75 mg/L hygromycin. Root and shoot lengths were measured after 7 days of incubation in the dark.

Seedlings of similar size were then transferred to fresh MS0 medium and grown under a 12 h light/12 h dark cycle at 65% humidity for an additional 7 days, after which they were measured again. Following 2 days of hardening, the seedlings were transplanted into 8-inch outdoor buckets filled with nutrient soil (black soil: vermiculite at a ratio of 3:1). Three buckets per genotype were cultivated, with 5 plants per bucket, for a total of 45 days.

#### 4.2.4. Observation of Plant Phenotypes During Vegetative Growth Stage

During the vegetative growth phase, measurements of plant height, the number of tillers per plant, and the content of ABA were taken in both CK and over-expression lines. Upon reaching maturity, the number of grains per panicle and the weight of the panicles were assessed. All data were collected using five biological replicates.

#### 4.2.5. Preparation of Semi-Thin Sections of Rice Leaves

Leaf segments (1 cm^2^) were excised from the middle of leaves, immersed in 2.5% glutaraldehyde (pH 7.0) in penicillin bottles, vacuum-infiltrated at 4 °C until sunken, and fixed overnight. Samples were then subjected to osmium tetroxide fixation, acetone gradient dehydration, infiltration, embedding, and polymerization. Ultrathin sections were cut using a Leica UC6 microtome, stained with 1% toluidine blue in borax, and observed under a microscope.

#### 4.2.6. Analysis of Relative Water Content in Rice Leaves

The third true leaf was harvested, and its fresh weight was recorded (M1). Subsequently, the leaf was submerged in double-distilled water in the dark for 24 h, blotted dry with filter paper, and reweighed to obtain the saturated fresh weight (M2). The leaf was then dried at 80 °C until it reached a constant dry weight (M3). The relative water content (RWC) was calculated using the following formula: RWC = (M1 − M3)/(M2 − M3) × 100%. Each measurement was replicated five times.

#### 4.2.7. Analysis of Water Loss Rate of Detached Rice Leaves

To determine the water loss rate of detached rice leaves, intact leaves were immersed in double-distilled water in the dark for 6 h. After blotting the leaf surfaces dry with filter paper, the saturated fresh weight (M1) was recorded. Subsequently, leaves were reweighed at 30, 60, 90, 120, and 150 min (Mn). The in vitro water loss rate was calculated using the following formula: (M1 − Mn)/M1 × 100% [29]. All measurements were performed in quintuplicate.

#### 4.2.8. Analysis of Chlorophyll Efflux Rate in Rice Leaves

Epidermal permeability was evaluated using the chlorophyll efflux assay. The third leaf from the top of each plant was excised, cut into 2 cm segments, and then immersed in 30 mL of 80% ethanol. The samples were kept in the dark at room temperature with gentle agitation. At each time interval, 2 mL of the solution was sampled for absorbance measurements at 664 nm and 647 nm using a DU640 UV/V is spectrophotometer in a dark room, after which the samples were returned to their original tubes. Chlorophyll concentrations were calculated following standard protocols [30]. Chlorophyll efflux at each time point was expressed as a percentage of the total chlorophyll extracted after 24 h. All measurements were conducted in triplicate.

#### 4.2.9. Analysis of ABA Content in Rice Leaves

Fresh leaf tissue (0.5–1.0 g) was weighed, snap-frozen in liquid nitrogen, and ground into a fine powder. The powder was then transferred to a 10 mL plastic centrifuge tube and sent to China Agricultural University for ABA content analysis.

#### 4.2.10. Preparation and Observation of Leaf Samples for Scanning Electron Microscopy

During vegetative growth, leaf samples from the same position of over-expression and CK plants were collected. The samples were fixed overnight in 2.5% glutaraldehyde at 4 °C, rinsed three times with 0.1 M sodium phosphate buffer, and post-fixed with 2% OsO_4_ at 4 °C. Subsequently, the samples underwent ethanol dehydration, amyl acetate infiltration, and critical point drying. Gold/palladium coating was performed using a JEOL JFC-1600 coater, after which wax crystals were observed, and stomatal aperture and width were measured under a JSM-7500F scanning electron microscope (JEOL, Tokyo, Japan).

#### 4.2.11. Analysis of Epidermal Wax and Cutin Extraction and Composition in Rice Leaves

During the vegetative growth phase, leaf samples were collected from the same position on both over-expression and the CK plants. The leaf areas were measured using ImageJ 1.53, after which the leaf waxes were extracted with n-hexane. The extracts were then concentrated to a volume of 5 μL/cm^2^ using HPLC-grade n-hexane and subsequently sent to China Agricultural University for GC-MS analysis to identify the wax and cutin components.

#### 4.2.12. Data Statistical Analysis

The experimental data were analyzed using SPSS 22.0 (Statistical Product and Service Solutions 22.0 for Windows). Significant differences among the various comparisons were determined using Duncan’s multiple range test and visualized with GraphPad Prism 8.

## 5. Conclusions

This study indicates that under normal conditions, transgenic rice lines over-expressing *AtNCED3* exhibited a 27.0% and 25.8% increase in cutin content and a 27.0% and 25.8% increase in wax content, respectively, compared to the control lines. SEM and GC-MS analyses revealed that the over-expression of *AtNCED3* altered the composition of wax and cutin monomers and modified the density of epicuticular wax crystals. Quantitative real-time PCR analysis demonstrated that the expression levels of nine wax-related genes and six cutin-related genes were significantly upregulated in the over-expressing lines. The relative water content of the over-expressing lines was 25.72% and 13.93% higher than that of the control lines, respectively. In vitro water loss rate experiments showed that the water loss rate of the over-expressing lines was lower within 150 min. Chlorophyll efflux experiments confirmed that the efflux rate was significantly reduced within 180 min. Consequently, the over-expressing lines displayed reduced epidermal permeability, lower transpiration rates, and enhanced drought resistance. These findings confirm that the over-expression of *AtNCED3* may be an effective strategy to enhance crop drought tolerance by increasing ABA levels, offering new insights for the development of drought-resistant crops.

## Data Availability

Data are contained within the article and Supplementary Materials..

## Supplementary Materials

The following supporting information can be downloaded at https://www.mdpi.com/article/10.3390/ijms26146690/s1.
